# *Twist1* Is a TNF-Inducible Inhibitor of Clock Mediated Activation of *Period* Genes

**DOI:** 10.1371/journal.pone.0137229

**Published:** 2015-09-11

**Authors:** Daniel Meier, Martin Lopez, Paul Franken, Adriano Fontana

**Affiliations:** 1 Institute of Experimental Immunology, University of Zurich, Zurich, Switzerland; 2 Center for Integrative Genomics, University of Lausanne, Lausanne, Switzerland; University of Lübeck, GERMANY

## Abstract

**Background:**

Activation of the immune system affects the circadian clock. Tumor necrosis factor (TNF) and Interleukin (IL)-1β inhibit the expression of clock genes including *Period* (*Per*) genes and the PAR-bZip clock-controlled gene *D-site albumin promoter-binding protein* (*Dbp*). These effects are due to cytokine-induced interference of E-box mediated transcription of clock genes. In the present study we have assessed the two E-box binding transcriptional regulators *Twist1* and *Twist2* for their role in cytokine induced inhibition of clock genes.

**Methods:**

The expression of the clock genes *Per1*, *Per2*, *Per3* and of *Dbp* was assessed in NIH-3T3 mouse fibroblasts and the mouse hippocampal neuronal cell line HT22. Cells were treated for 4h with TNF and IL-1β. The functional role of *Twist1* and *Twist2* was assessed by siRNAs against the *Twist* genes and by overexpression of TWIST proteins. In luciferase (luc) assays NIH-3T3 cells were transfected with reporter gene constructs, which contain a 3x*Per1* E-box or a *Dbp* E-box. Quantitative chromatin immunoprecipitation (ChIP) was performed using antibodies to TWIST1 and CLOCK, and the E-box consensus sequences of *Dbp* (CATGTG) and *Per1* E-box (CACGTG).

**Results:**

We report here that siRNA against *Twist1* protects NIH-3T3 cells and HT22 cells from down-regulation of *Period* and *Dbp* by TNF and IL-1β. Overexpression of *Twist1*, but not of *Twist2*, mimics the effect of the cytokines. TNF down-regulates the activation of *Per1-3xE-box-luc*, the effect being prevented by siRNA against *Twist1*. Overexpression of *Twist1*, but not of *Twist2*, inhibits *Per1-3xE-box-luc* or *Dbp-E-Box-luc* activity. ChIP experiments show TWIST1 induction by TNF to compete with CLOCK binding to the E-box of *Period* genes and *Dbp*.

**Conclusion:**

*Twist1* plays a pivotal role in the TNF mediated suppression of E-box dependent transactivation of *Period* genes and *Dbp*. Thereby *Twist1* may provide a link between the immune system and the circadian timing system.

## Introduction

Clock genes mediate circadian rhythmicity and thereby control behavior, sleep-wake cycles, metabolism and inflammation [1–3]. Synchronization of the cellular clocks in the body is coordinated by the suprachiasmatic nuclei (SCN) in the brain. At the molecular level the clock consists of a network of transcription factors that functions in self regulated transcription-translation feedback loops. The heterodimerized transcription factors CLOCK-BMAL1 (brain and muscle ARNT-like protein) activate transcription by binding to the cis-regulatory element E-box in the promoters of *Period* (*Per*), *Cryptochrome* (*Cry*), and of the members of the PAR-bZip family of transcription factor genes including the *D-site albumin promoter-binding protein (Dbp)* [4, 5]. PER and CRY are transported into the cell nucleus where they inhibit the transcriptional activity of CLOCK-BMAL1 complexes, thereby inhibiting their own gene expression. The same positive and negative regulatory components also govern the rhythmic expression of the nuclear orphan receptor *Rev-Erb-alpha*, which in turn represses the transcription of *Bmal1*. These pathways drive the 24-h rhythms in physiology.

Sickness behavior syndrome (SBS) with fatigue and depression is observed in states of immune activation in the course of infections and autoimmune diseases [6]. SBS can be induced by peripheral or central administration of proinflammatory cytokines. The effect of the cytokines is associated with altered expression of clock genes. TNF and IL-1β have been shown to lead to inhibition of expression of *Per1*, *Per2*, *Per3*, *Cry1* and *Cry2* and of the PAR-bZip clock- controlled genes *Dbp*, *tyrotroph embryonic factor* (*Tef*), and *hepatic leukemia factor* (*Hlf*) [7]. In NIH-3T3 fibroblasts TNF exerts its effect by inhibiting CLOCK-BMAL1 induced activation of E-box regulatory elements in *Dbp* and *Per* promoters [7]. Interference with clock gene expression is also mediated by transforming growth factor beta (TGFβ) [8, 9]. TGFβ is overexpressed in neurons in Alzheimer`s disease and has been proposed to alter the circadian rhythm in aged patients [9].

The mechanisms involved in dysregulation of clock genes by immune activation have just recently been addressed. Because the addition of TNF to NIH-3T3 cells was found to increase *Per* and *Cry* genes in the first three hours before exerting its inhibitory effects in the following hours, it was thought that TNF triggers the well described negative feedback loop by which PER-CRY inhibit their own transcription. Experiments using *Per1*,*Per2* ^-/-^ and *Cry1*,*Cry2* ^-/-^ mouse embryonic fibroblasts did not confirm this hypothesis [10]. Another approach made use of recent studies, which indicate that the cold-inducible RNA-binding protein (CIRBP) is required for high amplitude circadian gene expression [11]. Among the transcripts interacting with CIRBP are the mRNAs encoding *Per2*, *Per3*, and *Dbp*, which were decreased in *Cirbp*-depleted cells [11, 12]. TNF was found to modulate clock gene expression by impairing the production of CIRBP [13]. However lower *Cirbp* expression was only responsible for the effect of TNF when using suboptimal TNF concentrations in the NIH-3T3 cell assay. Taken collectively the pathways involved in TNF mediated abnormal clock gene expression are still unknown. This contrasts the inhibitory effect of TGFβ on clock genes, which results from the induction of *Dec1* [8]. This E-box binding protein competes with CLOCK:BMAL1 for the DNA binding in clock genes.

In the search for factors, which upon induction by TNF interfere with E-box mediated transcription we have put our focus on the transcriptional regulators *Twist 1* and *Twist 2*. The two members of the basic helix-loop-helix (bHLH) family of proteins have been characterized as transcriptional regulators, which bind to E-boxes [14]. TWIST2 is 66% identical to TWIST1 and the identity increases to 98% in the basic and HLH regions of the proteins. At the C-terminus a repressor domain termed “Twist box” is located, which is identical in TWIST1 and TWIST2. However, the two DNA binding proteins differ in their individual roles as activators and repressors of gene transcription. *Twist1* is expressed in embryonic stages and is important for early development and osteoblast differentiation [15, 16]. Recent work also shows that *Twist1* is overexpressed in many human cancers and cancer cell lines, thereby driving tumor invasion and metastasis [17–19]. TWIST1 and -2 are known to repress cytokine gene expression and epithelial–mesenchymal transition through interaction with NFκB mediated transcription [20]. Both proteins form dimers and depending on their phosphorylation state may interact with other bHLH family members including the E-proteins E12/E47 [14].

The property of TNF to suppress the expression of clock genes may involve TWIST1 and TWIST2. This hypothesis is based on (i) the effect of TNF to interact with the *Clock / Bmal1* induced activation of E-boxes of *Period* genes and *Dbp* [7], (ii) the property of TWIST1 and TWIST2 to bind to E-boxes, and (iii) on the induction of *Twist1* and *Twist2* by TNF, the effect being mediated by NF-κB [20, 21]. The hypothesis of Twist genes being involved in TNF mediated clock gene inhibition is supported by the recent finding of *Twist1* and *Twist2* to inhibit the expression of TNF, which may indicate bidirectional regulatory control of their expression [20]. Here, we describe the novel function of *Twist1* to serve as a transcriptional repressor mediating TNF effects on *Period* and *Dbp* genes by interfering with CLOCK induced E-box binding and functions thereof.

## Materials and Methods

### Cell culture

The murine fibroblast cell line NIH-3T3 (CRL-1658) was obtained from the American Type Culture Collection. The mouse hippocampal neuronal cell line HT22 was obtained from David Schubert at the Salk Institute (La Jolla, CA). [22]. NIH-3T3 cells and HT22 cells were grown in DMEM (Gibco) with 10% FBS (Sigma) at 37°C and 5% CO_2_. For assessing the effect of Twist on clock genes cells were synchronized for 1h with serum deprivation and then treated with TNF (0.1, 1.0 or 10ng/ml), IL-1β (0.1, 1 or 10ng/ml) or TGFβ2 (2ng/ml) (Peprotech) for 4h as described recently [7]. Time kinetic analysis was performed by treating the cells with TNF (10ng/ml) for 24hr, cells being harvested every three hours.

### SiRNA experiments and overexpression

Knockdown experiments were performed in NIH-3T3 and HT22 using ON-TARGETplus siRNA (Dharmacon) against *Twist1* and a DharmaFECT transfection reagent. Twenty-four hours before transfection, the cells were seeded in 6-well plates (150,000 cells/well). The transfections used siRNA solutions (5 μM) in 1× siRNA buffer (Dharmacon). The transfection reactions were performed according to the manufacturer's instructions. After 48 h, the cells were harvested for mRNA analyses.

Overexpression of *Twist1* and *Twist2* was established by stable transfection of TrueClone pCMV6::Twist1, pCMV6::Twist2 and pCMV6::empty plasmid (Origene) into NIH-3T3 cells with FugeneHD transfection reagent (Promega) according to the manufacturer's instructions. G418 (Promega) was used as selection antibiotics at a concentration of 0.5mg/ml.

### RNA extraction, cDNA synthesis and qRT-PCR analyses

Total RNA from NIH-3T3 and HT22 cells were obtained with the NucleoSpin RNAII Kit (Macherey-Nagel), including DNase digestion. Total RNA (1 μg), anchored oligo(dT)_18_ primer (2.5 μM) and reverse transcriptase (10 U) from the nanoScript 2 reverse transcription kit (PrimerDesign) were used for the first strand synthesis according to the manufacturer's suggestions. The cDNA was stored at −20°C.

The qRT-PCR primers for *Twist1*, *Twist2*, *Dbp*, *Per1*, *Per2* and *Per3* were commercially available from PrimerDesign Each of the samples was examined in triplicate and subjected to qRT-PCR using PrecisionPLUS qPCR Mastermix (PrimerDesign). *HPRT* and *eEF1a1* probes were used as internal controls. Relative RNA expression levels were determined by normalizing to the internal controls, and the values were calculated using the comparative Ct method [23].

### Western blot analysis

Immunoblots were performed with samples containing total protein (40 μg) and 12% NuPage Bis-Tris or 7% Tris-Acetat polyacrylamide gels (LifeTechnologies). The membranes were probed with rabbit polyclonal anti-PER1 (1:500; Abcam ab136451), rabbit polyclonal anti PER2 (1:250; ab180655), rabbit monoclonal anti-PER3 (1:2500; ab177482), rabbit polyclonal anti-DBP (1:400; ab22824), rabbit polyclonal anti-Twist1 (1:400; ab49254) and rabbit polyclonal anti-TWIST2 antibodies (1μg/ml; ab66031). The secondary antibody was a horseradish peroxidase-linked goat anti-rabbit IgG (various concentrations; ab97051), and it was detected by the chemiluminescence technique using the Immobilon Western ECL system (Millipore). For a loading control, we used a mouse monoclonal anti-p84 antibody (nuclear matrix protein 84; 1:2000; Abcam ab487). The secondary antibody was a horseradish peroxidase-linked goat anti-mouse IgG (1:5000; Abcam; ab20043). Densitometric analysis was performed using the ImageJ software and the intensity of the control lanes were set as 1.

### Cotransfection and dual luciferase assay

NIH-3T3 cells were grown as described above. The transfections were performed with Effectene transfection reagent (Qiagen) according to the manufacturer's protocol. Briefly, 24 h before transfection, cells were seeded in 12-well plates (200,000 cells/well). The transfections required DNA (450 ng), the pGL3 promoter vector (firefly) with our inserts, the pRL SV40 vector (*renilla*) as an internal control, and 240.8 μl of transfection reagent for triplicates (212 μl of EC Buffer, 10.8 μl of Enhancer and 18 μl Effectene). At 48 h after transfection, cells were washed with PBS and lysed with passive lysis buffer (Promega) to perform a conventional dual luciferase assay (DLR). Lysate (20 μl) was placed into each well of a white 96-well plate and measured with an Infinite M200 pro Luminometer (Tecan). The DLR was performed according to the manufacturer's instructions, except that the amount of substrate was reduced to 50 μl per aliquot. In this assay we tested first a construct containing 3 E-boxes from the *Period1* promoter (TTTAGC*CACGTG*ACAGTGTAAGCA*CACGTG*GGCCCTCAAGTC*CACGTG*CAGGGA), as well as the mutated counterpart (TTTAGC*ACCGGT*ACAGTGTAAGCA*ACCGGT*GGCCCTCAAGTC*AC* *CG* *GT*CAGGGA). From the *Dbp* promoter one E-box, wild type (CACGT*CCCATG*TGGCC) and mutated (CACGT*C* *G* *CA* *GT*TGGCC) was cloned into the reporter plasmid according to: [24, 25]. The *Period1* E-Box, as well as the mutated sequence were inserted into the pGL3 promoter vector via the *Nhe1/Xho1* site. The cloned sequence consists of three E-boxes within 2.0 kb of the 5’-flanking region of the mouse *Per1* gene. The *Dbp* E-Box and the mutated counterpart were amplified out of a shuttle plasmid and ligated into the *BglII* restriction site of the pGL3 promoter vector.

### Chromatin immunoprecipitation (ChIP)

NIH-3T3 cells were grown in 15 cm petri dishes under normal conditions up to 80% confluency and fixed with formaldehyde. Fixation, enzymatic shearing and immunoprecipitation were performed with the ChIP-IT Express Enzymatic Kit (Active Motif) according to the manufacturer’s instructions. Chromatin was precipitated with either a monoclonal ChIP grade antibody against TWIST1 (Abcam, ab50887) or anti CLOCK antibody (ab 3517). As positive control a monoclonal RNA Pol II antibody, as negative control a IgG antibody was used 10μg of sheared chromatin and 2μg of antibody was used in 100μl reaction volume. Eluted and reverse cross-linked chromatin was analyzed via qPCR of region of interest. Primers for E-box regions:

*Per1* E-box:

fw: 5’-GGGTAGTTTCCCTCCCTCAC-3’

rv: 5’- TGGCATCTGATTGGCTACTG-3’.

*Dbp* E-box:

fw: 5’-TCAACATGGTACAGCCCAGA-3’

rv: 5’-TGTGGGAGCTGAGCACATAG-3’.

The analysis method entailed solving for the DNA quantity of the ChIP and IgG samples, then calculating the fold enrichment of the ChIP sample relative to the IgG sample.

### Statistics

All experiments were performed in triplets and analyzed with GraphPad Prism (GraphPad Software). To assess the statistical significance between single series of measurements (i.e. untreated vs. treated) we used the two-tailed Student’s *t*-test. For grouped measurements with one variable for the selected genes, an one-way ANOVA was used, followed by a Bonferroni post-hoc test to decrease the likelihood of a rare event in multiple comparisons. In the case of two variables (i.e. cytokine treatment and siRNA/overexpression) a two-way ANOVA was used to measure the statistical significance, also followed by a Bonferroni post-hoc test to decrease the α-error margin. *P* values less than 0.05 were considered significant, 0.01 in ANOVAs with post-hoc tests. +/- SEM is shown by error bars.

## Results

**Fig 1:**
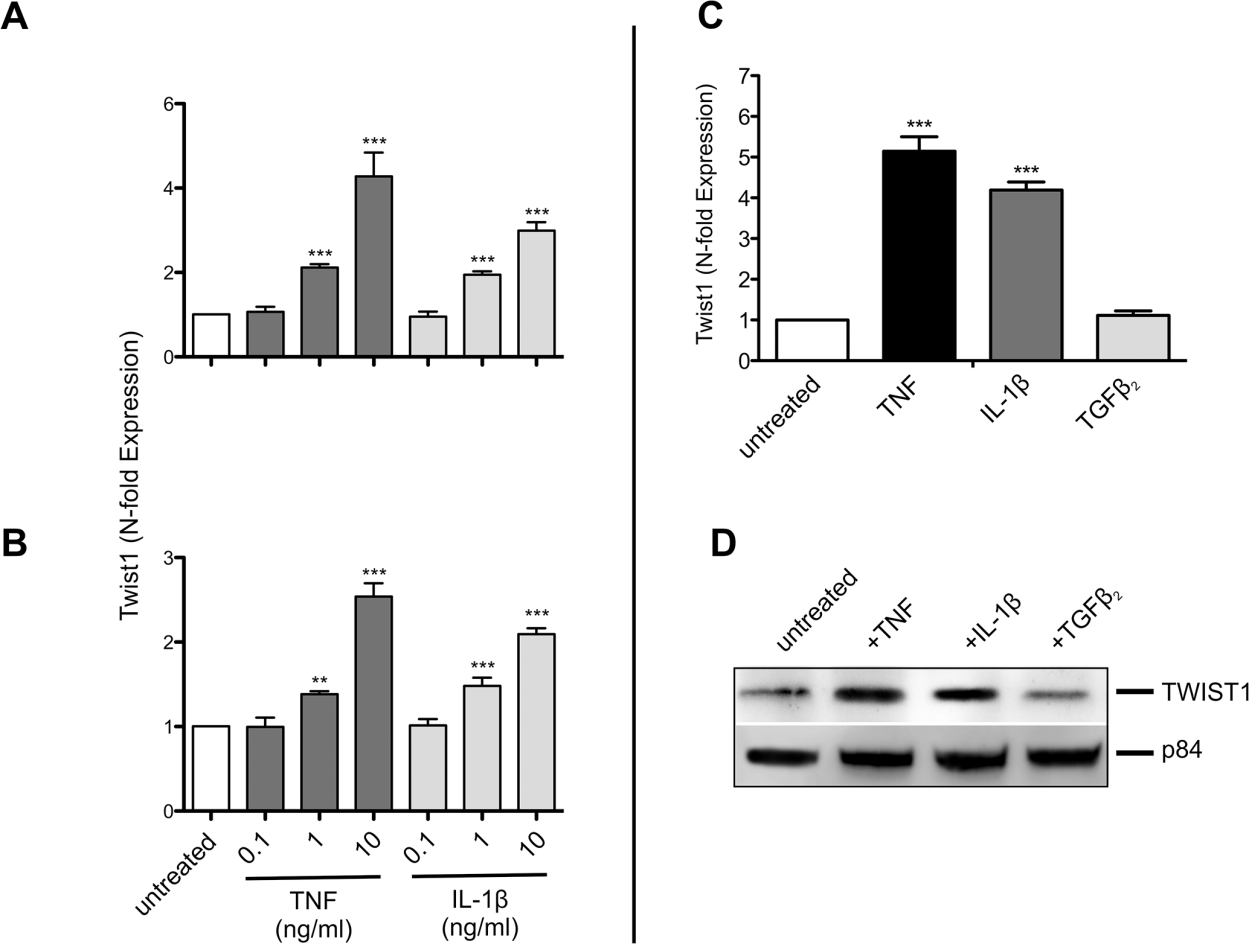
*Twist1* is induced by TNF and IL-1β, but not by TGFβ2. Dose dependent expression of *Twist1* mRNA in NIH-3T3 (A) and HT22 (B) cells after a 4 hr treatment with different concentrations of TNF (0.1, 1 and 10ng/ml), and IL-1β (0.1, 1 and 10ng/ml). (C) Expression of *Twist1* mRNA in NIH-3T3 after a 4 hr treatment with TNF (10ng/ml) and IL-1β (10ng/ml). The dose of TGFβ2 added (2ng/ml) was chosen from previous studies on effects of this cytokine on the expression of clock genes [8, 9]. (D) Western blot showing upregulation of TWIST1 in cell lysates of NIH-3T3 cells treated for 4 hr with TNF (10ng/ml), IL-1β (10ng/ml), but not after TGFβ2 treatment (2ng/ml). Nuclear matrix protein p84 was used as loading control. Data of RT-qPCR assays show the mean +/- SEM (error bars) of triplicates from three independent experiments. Significance of grouped results were calculated with one way-ANOVA and Bonferroni post-hoc test;*** p<0.001. Western blot shows one representative experiment of three.

Recent studies showed TNF and IL-1β to increase the expression of *Twist* genes in various types of cells [21, 26]. To test whether NIH-3T3 fibroblasts and HT22 hippocampal neuronal cells similarly respond to cytokines with upregulation of *Twist1* cells were treated for 4 h with TNF and IL-1β at various concentrations. Both TNF and IL-1β induced the expression of *Twist1* in a dose dependent fashion (Fig 1A and 1B). In contrast to TNF and IL-1β, TGFβ2 failed to augment the expression of *Twist1* mRNA in NIH-3T3 cells (Fig 1C). Upregulation of TWIST1 by TNF and IL-1β, but not by TGFβ2 was also seen at the protein level (Fig 1D). Densitometric analysis of the Western blot showed TNF and IL-1β to increase TWIST1 and TGFβ2 to have only a minor inhibitory effect. Compared to untreated cells TWIST1 expression in cells treated with the aforementioned cytokines was 3.3, 2.8 and 0.9-fold respectively.

**Fig 2:**
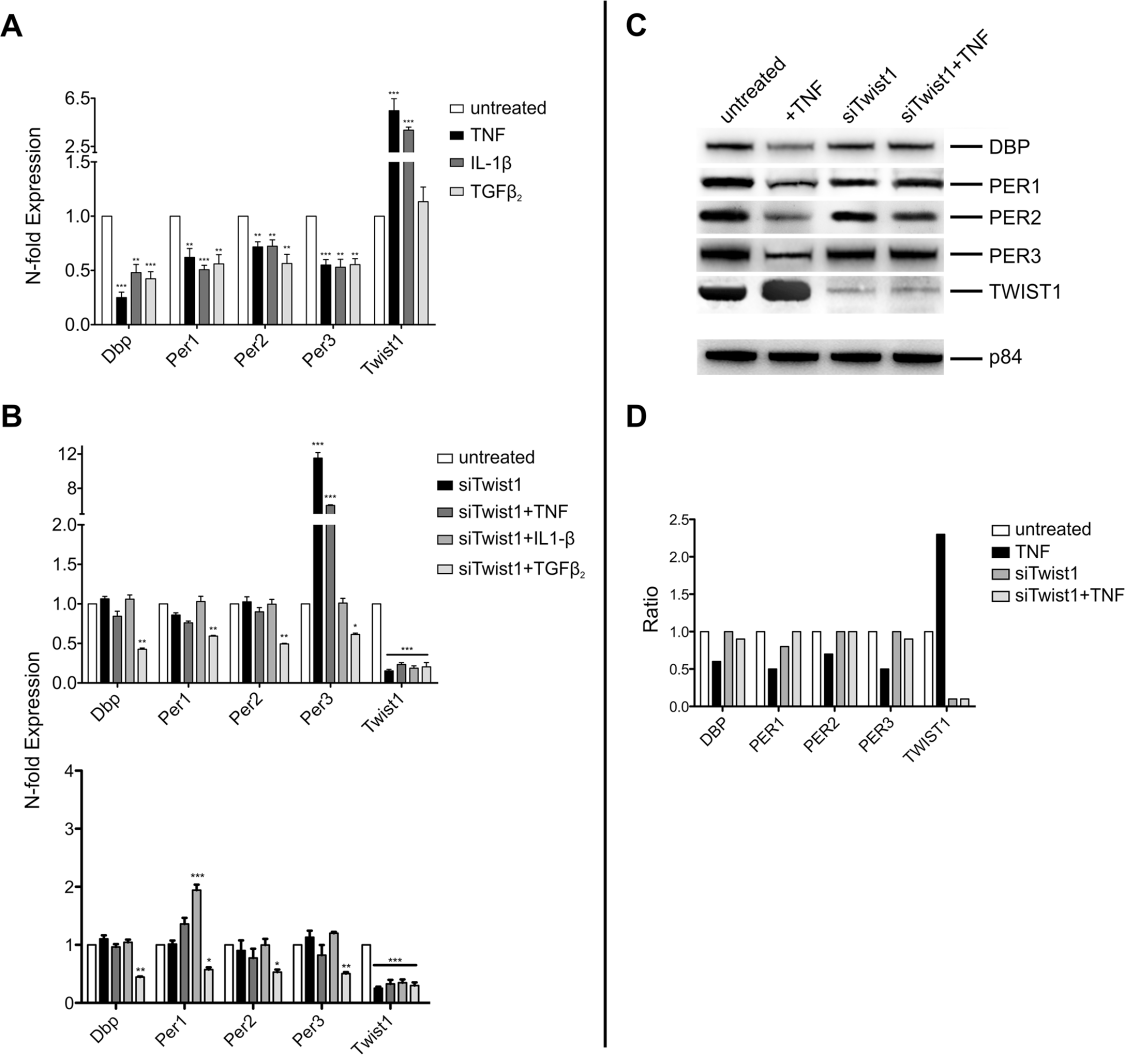
siRNA against *Twist1* prevent from inhibition of clock gene expression by TNF and IL-1β. (A) Expression of clock genes in NIH-3T3 cells treated with TNF (10 ng/ml), IL-1β (10 ng/ml) and TGFβ2 (2 ng/ml) for 4 hr. Data of RT-qPCR assays of *Dbp*, *Per1*, *Per2*, *Per3*, and *Twist1* mRNA expression show the mean +/- SEM (error bars) of triplicates from three independent experiments. (B) Effect of cytokines on clock genes in NIH-3T3 cells (upper panel) and HT22 cells (lower panel), the cells being pretreated with siRNA against *Twist1*. *(siTwist1* **).** (C) Western blot of lysates from NIH-3T3 cells, which were pretreated with siRNA against *Twist1* and thereafter with TNF for 4 hr. Matrix protein p84 was used as loading control. (D) Intensity of bands, which are shown in Fig 2C by Western blot. The data are given as ratio of untreated versus treated cells. Untreated lanes were set as 1. The concentrations of the cytokines used in the experiments are given in legend to Fig 1C. Significance of RT-qPCR data was calculated with two way-ANOVA and Bonferroni post-hoc test;* p<0.01, ** p<0.01, *** p<0.001. Western blot shows one representative experiment of three.

Cytokines have been shown to interfere with the expression of clock genes [7–9]. Consistent with these reports we find a 4h treatment of NIH-3T3 cells with TNF and IL-1β to inhibit the expression of *Dbp*, *Per1*, *Per2*, and *Per3* (Fig 2A). While having no effect on *Twist1* expression, TGFβ2 shared with TNF and IL-1β its effects on clock genes. Next we determined whether *Twist1* induction is responsible for the down-regulation of clock genes by cytokines. The hypothesis was tested by suppressing *Twist1* expression by siRNA. NIH-3T3 were transfected for 36 h with siRNA against *Twist1* and siNOtarget control. Depletion of *Twist1* resulted in an almost complete prevention of the TNF and IL-1β mediated suppression of *Dbp*, *Per1*, *Per2* and *Per3* (Fig 2B). However, siRNA against *Twist1* did not alter the inhibitory effect of TGFβ2 on the expression of clock genes. Whereas siRNA against *Twist1* had no effect on basal expression of *Dbp*, *Per1* and *Per2*, it upregulated the expression of *Per3* irrespective of whether the cells were exposed to TNF or not (Fig 2B). Moreover in NIH-3T3 cells treated with siRNA against *Twist1* the upregulation of *Per3* was not seen in the presence of IL-1β or TGFβ2. To assess whether the prevention of cytokine mediated inhibition of *Period* genes and of *Dbp* by siRNA against *Twist1* is unique to NIH-3T3 cells we studied another type of cell. As observed in NIH-3T3 cells also HT22 neuronal cells respond to TNF with up-regulation of *Twist1* (Fig 1B); prevention of this effect by siRNA against *Twist1* abolished the inhibitory effect of TNF on *Period* genes and on *Dbp* (Fig 2B). siRNA against *Twist1* up-regulated the expression of *Per3* only in NIH-3T3 cells, but not in HT22 cells (Fig 1B). The latter type of cell, however, responded to siRNA against *Twist1* with an up-regulation of *Per1* when exposed to IL-1β (Fig 2B). Taken collectively siRNA against *Twist1* prevents from TNF and IL-1β induced suppression of *Period* genes and *Dbp* in both NIH-3T3 and HT22 cells. The mechanisms, which lead to different effects on expression of *Per1* and *Per3* in the two types of cells treated with siRNA against *Twist1* are not yet clear.

When assessing the effect of TNF on clock genes at the protein level, treatment of NIH-3T3 cells with TNF was found to reduce the amount of DBP, PER1, PER2 and PER3 in NIH-3T3 cells (Fig 2C and 2D). The inhibitory effect of TNF on clock proteins was prevented by siRNA against *Twist1*. The increase of expression of *Per3* mRNA in cells treated with siRNA agaist *Twist1* was not observed at the protein level (Fig 2C and 2D).

**Fig 3:**
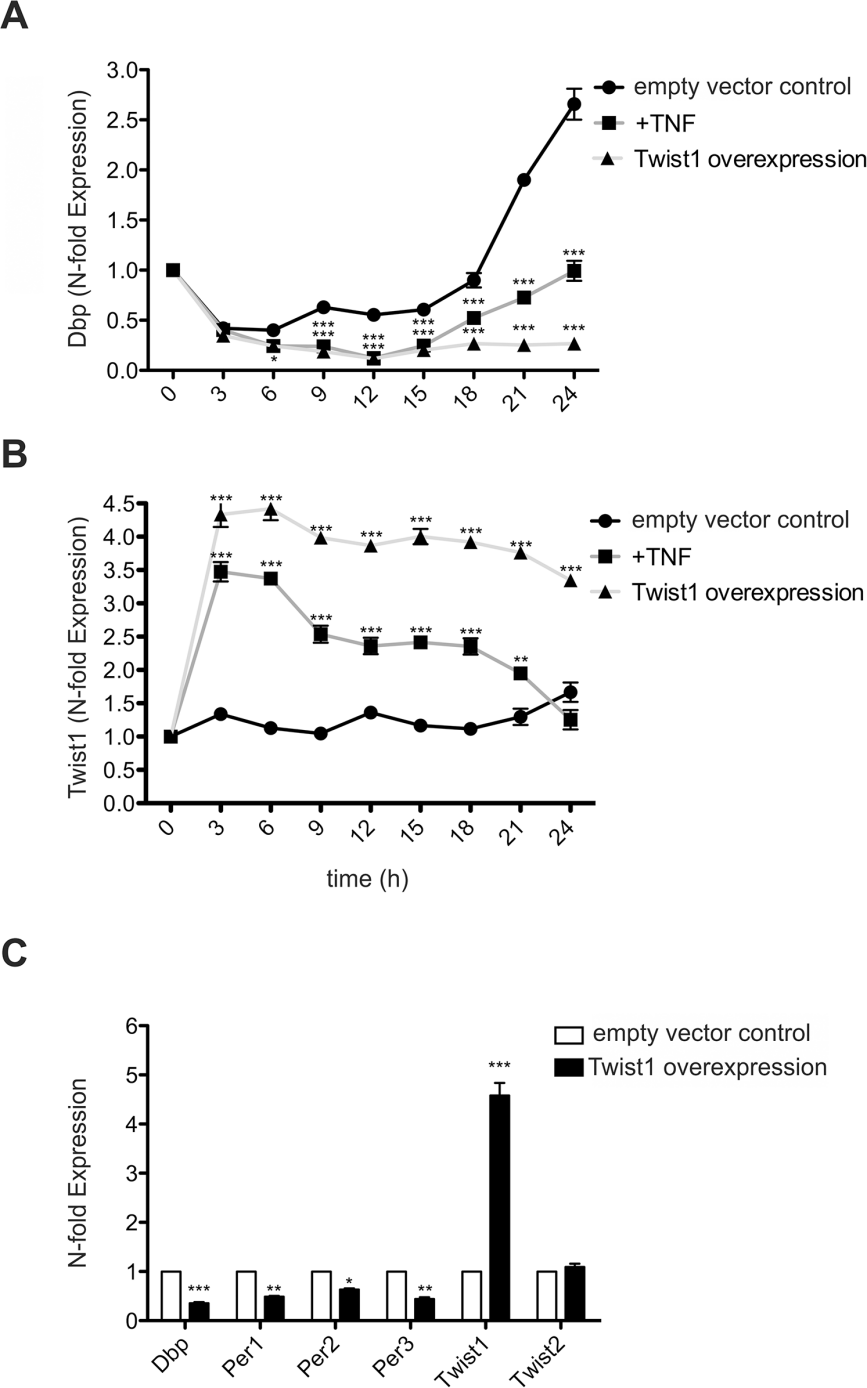
Time kinetic of TNF effects on *Twist1* and *Dbp* expression, and suppression of *Dbp* by overexpressiom of *Twist1*. (A) Expression of *Dbp* and (B) of *Twist1* mRNA in NIH-3T3, which were treated with TNF (10 ng/ml) for 3 to 24 hr or transfected with the pCMV6::*Twist1* expression vector. The control consisted of transfection with the empty vector (pCMV6). (C) Expression of clock gene mRNA after transfection with pCMV6::*Twist1* expression vector or empty vector control in NIH-3T3 cells. Overexpression of *Twist1* was controlled by RT-qPCR and the effect on *Twist2* assessed at 4hr. Data of RT-qPCR assays of *Dbp*, *Per1*, *Per2*, *Per3*, and *Twist1* mRNA expression show the mean +/- SEM (error bars) of triplicates from three independent experiments. Data are analyzed with one way-ANOVA and Bonferroni post-hoc test; * p<0.01, ** p<0.01, *** p<0.001

Next we performed time kinetic experiments using NIH-3T3 cells. As reported the NIH-3T3 cell line used in our study responds to serum deprivation with a rhythmic expression of *Dbp* mRNA; peak expression is observed at 24 hr (Fig 3A) [7]. Inhibition of *Dbp* mRNA by TNF was significant at all time points assessed. The induction of *Twist1* by TNF was maximal at 3hr and declined thereafter. Decreased expression of *Twist1* was paralleled by an increase of *Dbp* mRNA (Fig 3A and 3B). To determine the potential of *Twist1* to act as a repressor of clock genes we overexpressed *Twist1* and assessed the expression of clock genes in the absence of exogenously added cytokines. Overexpression of *Twist1* interfered profoundly with *Dbp* expression during the 24 hr study period (Fig 3A). The effectiveness of *Twist1* transfection using pCMV6 vectors was controlled and found to result in an increase of *Twist1* mRNA between 3.5 and 4.5 fold above the empty vector control (Fig 3B). As shown in Fig 3C NIH-3T3 cells, which overexpress *Twist1* for 4 hr show not only a reduction of *Dbp*, but also of *Per1*, *Per2* and *Per3* expression when compared to cells transfected with empty vector control. Overexpression of *Twist1* did not alter the expression of *Twist2* mRNA (Fig 3B). Taken collectively our data characterize *Twist1* as a repressor of clock genes.

**Fig 4:**
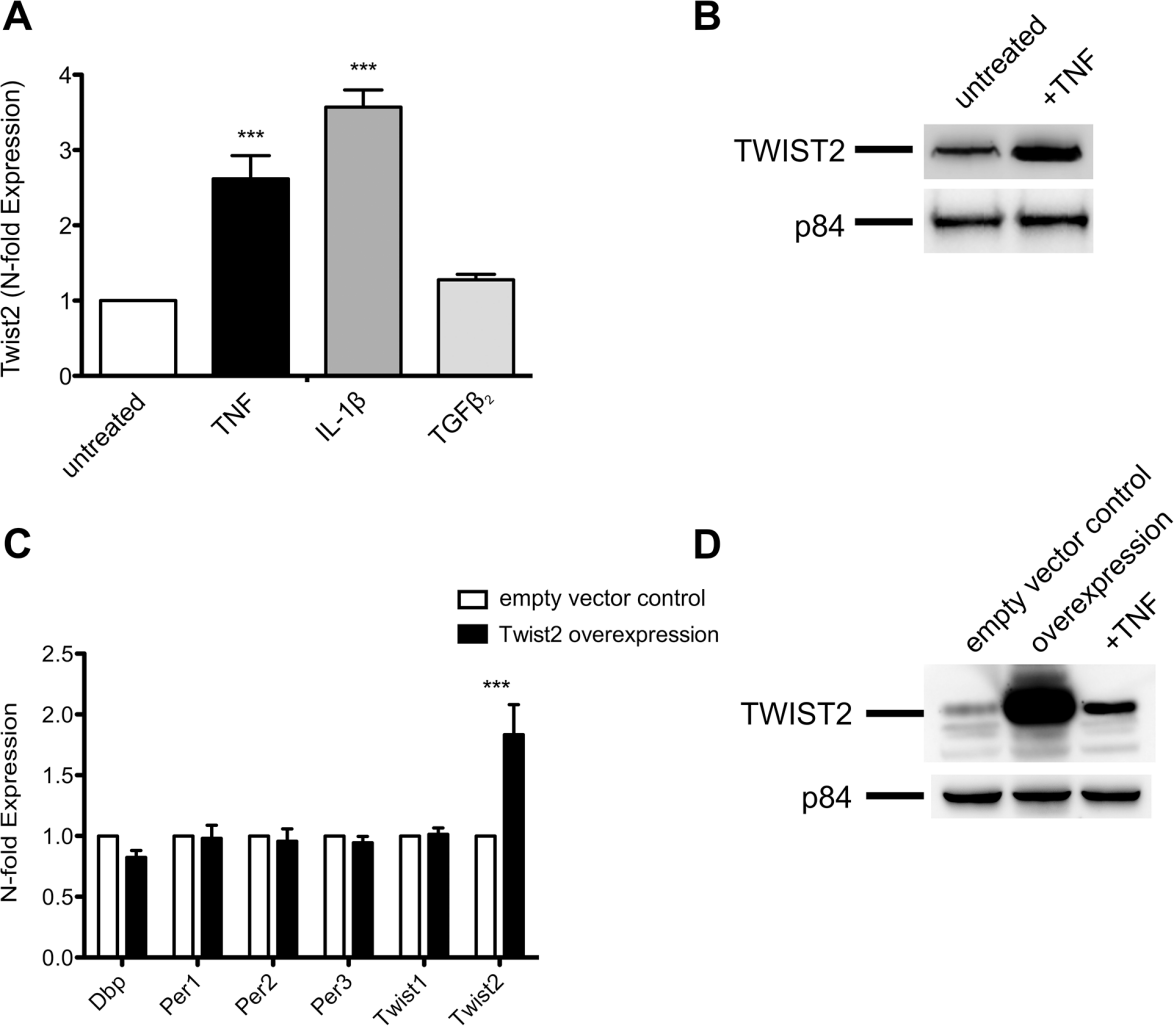
*Twist2* does not impair the expression of clock genes. (A) Expression of *Twist2* mRNA in NIH-3T3 cells after a 4 hr treatment with TNF, IL-1β and TGFβ2. The concentrations of the cytokines used are given in the legend to Fig 1. Data of RT-qPCR assays show the mean +/- SEM of three independent experiments. (B) Western blot showing upregulation of TWIST2 in cell lysates of NIH-3T3 cells treated with TNF for 4 hr. Nuclear matrix protein p84 was used as loading control. (C) Transfection of NIH-3T3 cells with pCMV6::*Twist2* show no significant effect on expression of *Dbp*, *Per1*, *Per2* and *Per3* compared to the empty vector control. (D) Data show TWIST2 expression in untreated (empty vector control) and TNF treated NIH-3T3 cells and in cells transfected with pCMV6::*Twist2*. Data of RT-qPCR assays show the mean +/- SEM (error bars) of triplicates from three independent experiments. Significance was calculated with two-way ANOVA and Bonferroni post-hoc test; *** p<0.001. Western blots show one representative experiment of three.

In the light of the high degree of homology of *Twist1* with *Twist2* we studied the effect of Twist2 on clock genes. As observed with *Twist1*, also *Twist2* mRNA and protein are upregulated by treatment of NIH-3T3 cells with TNF and IL-1β, but not when adding TGFβ2 to the cells (Fig 4A and 4B). The overexpression of TWIST2 failed, however, to interfere with the expression of clock genes (Fig 4C). Overexpression of TWIST2 was verified by Western blot (Fig 4D). Taken collectively we find that Twist2 does not share Twist1‘s inhibitory effects on clock genes.

**Fig 5:**
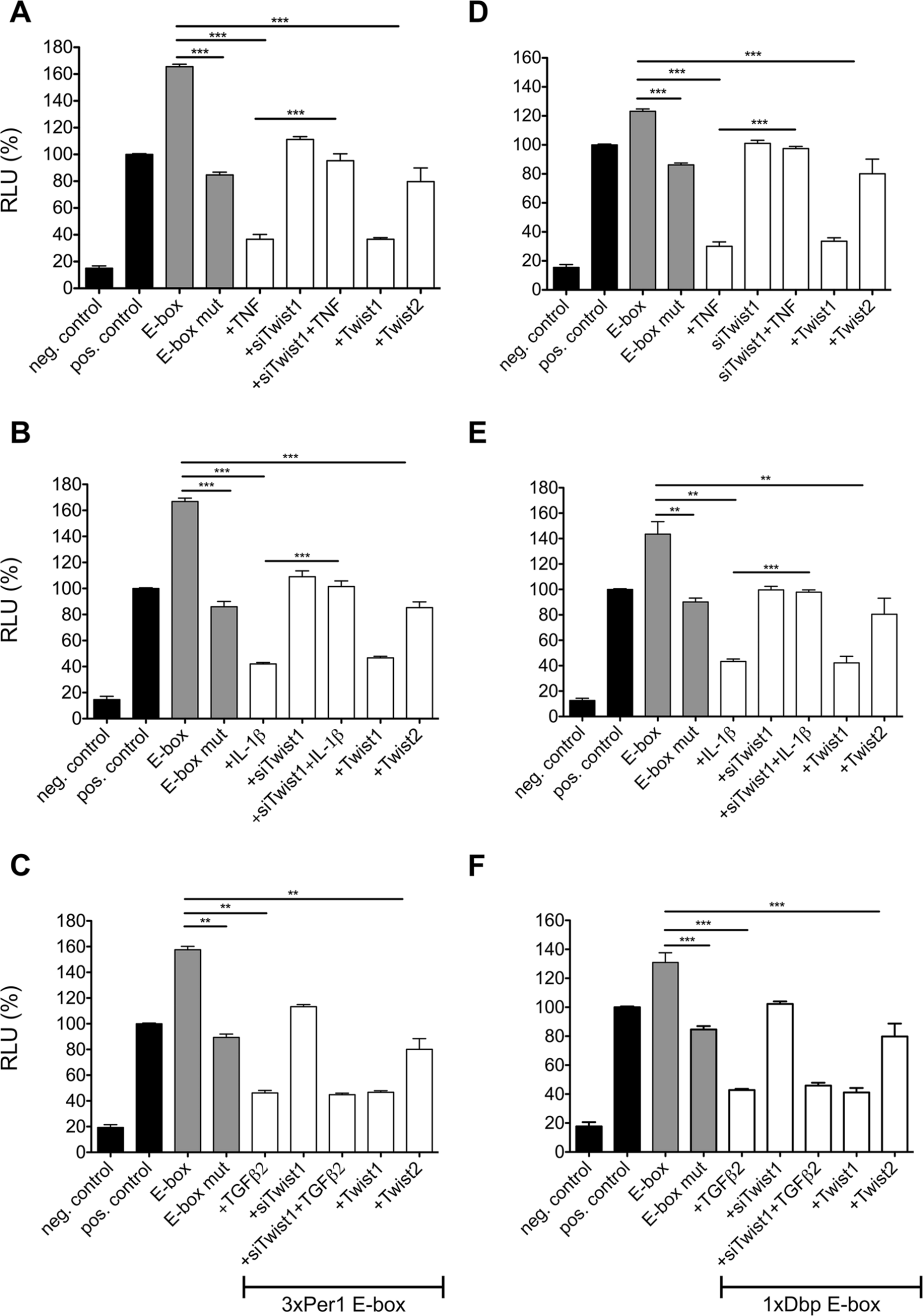
Dual luciferase assay for different E-box containing constructs. NIH-3T3 cells were transfected with different reporter gene constructs, which contain a 3x*Per1* E-box (A-C) or a *Dbp* E-box (D-F). The concentrations of the cytokines used are provided in the legend to Fig 1. Results are given for wild type and mutated E-box, as well as cytokine treated cells (4 hr) and co-transfection with *Twist1 and Twist2* overexpression plasmids respectively. *pGL3 basic* was used as negative, *pGL3* promoter as positive control. White bars represent the constructs containing the corresponding E-box. Data of RT-qPCR assays show the mean +/- SEM (error bars) of triplicates from three independent experiments. ** p<0.01, *** p<0.001; two-way ANOVA followed by the Bonferroni post-hoc test (n = 3)

E-boxes are functionally important components of DNA promoters that guide the expression of clock genes. Rhythmic binding of CLOCK and BMAL1 depends on E-boxes and is a prerequisite for robust waves of gene expression characteristic of circadian transcription [27, 28]. Previous data show that only clock genes with E-boxes in their promoter, the PAR bZip genes *Dbp*, *Tef*, and *Hlf* and the *Per* genes, are affected by TNF, whereas clock genes devoid of E-boxes such as *Clock* and *Bmal1* are not affected by TNF [7]. To explore the predication that *Twist1* operates at the site of E-elements in clock promoters we tested the effect of cytokines on *Per1-3xE-box-luciferase* (luc) reporter in the presence or absence of siRNA against *Twist1*. TNF down-regulates the activation of *Per1-3xE-box-luc* (Fig 5A). This effect of TNF was not observed when cells were transfected with siRNA against *Twist1*. On the other hand inhibition of TNF mediated *Per1-3xE-box-luc* activity was mimicked by overexpression of *Twist1*, but not of *Twist2*. An analogous picture emerged when testing IL-1β. The IL-1β induced suppression of *Per1-3xE-box-luc* was found to depend on the presence of *Twist1* (Fig 5B). However, depletion of *Twist1* by siRNA failed to prevent the inhibitory effect of TGFβ2 on *Per1-3xE-box-luc* activity (Fig 5C). The experiments using cytokines and siRNA against *Twist1* were also performed using *Dbp-E-Box-luc*. Confirming the experiments with the *Per1-3xE-box-luc* construct, the TNF and IL-1β induced inhibition of *Dbp-E-box-luc* activity was dependent on *Twist1* (Fig 5D and 5E). Again the effect of TGFβ_2_ was independent of the presence or absence of *Twist1* (Fig 5F). This finding is consistent with the failure of TGFβ2 to enhance the expression of *Twist1*.

**Fig 6:**
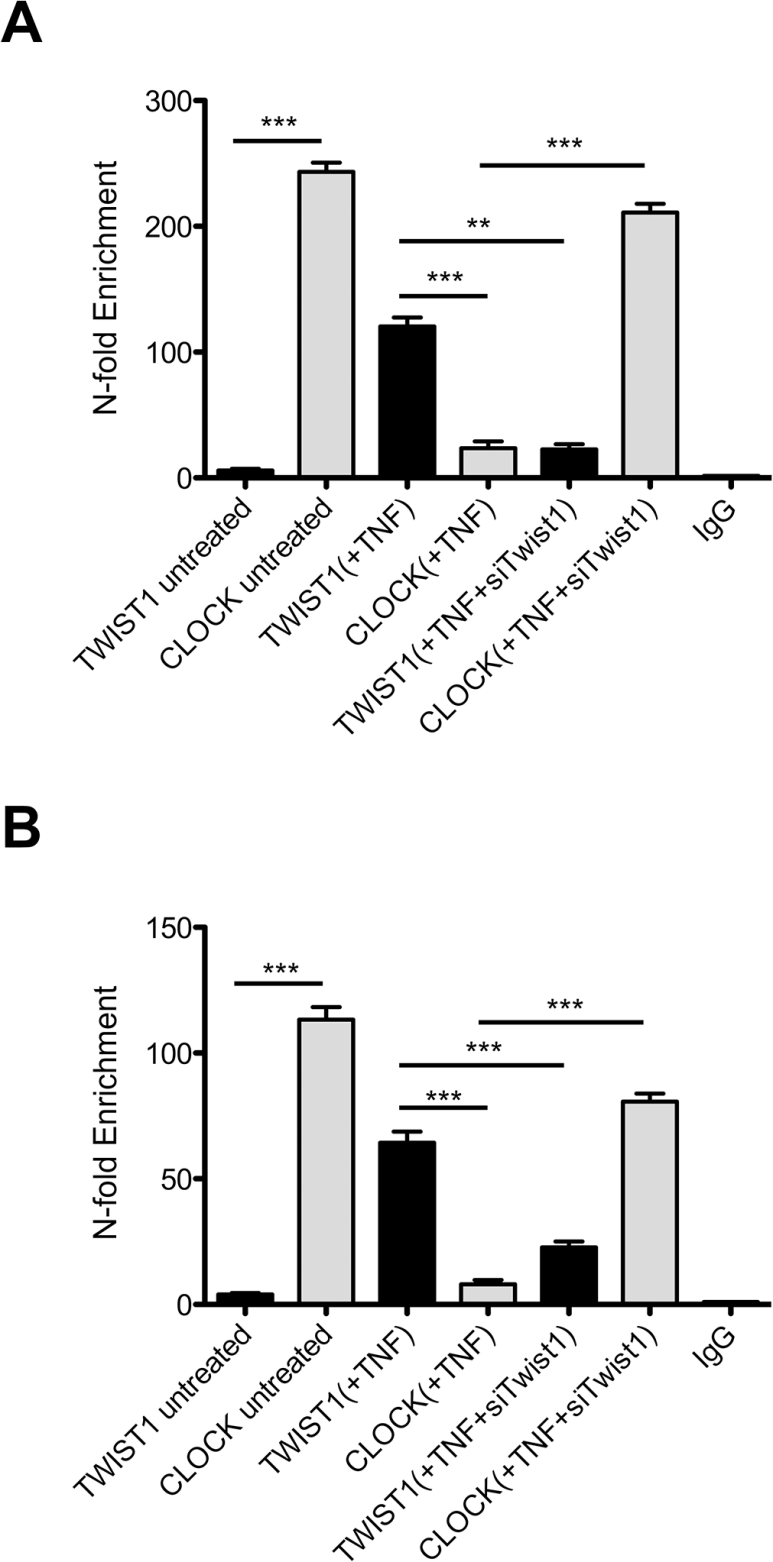
Quantitative ChIP for TWIST1 and CLOCK showed a preference of TWIST1 binding to E-box consensus sequence in the presence of TNF. Binding of TWIST1 and CLOCK on the *Dbp* (CATGTG) E-box (A) and *Per1* E-box (CACGTG) (B) in NIH-3T3 cells. The correspondent treatment is given in brackets. TNF (10ng/ml) treatment was performed for 4 hr. IgG antibody was used as negative control. Data of RT-qPCR assays show the mean +/- SEM (error bars) of triplicates from three independent experiments. The significance was determined by student’s t-test; ** p<0.01, *** p<0.001.

To test whether the E-box of *Dbp* and *Per1* are transcriptional targets of TWIST1, we performed quantitative ChIP experiments using anti-TWIST1 and anti-CLOCK antibodies. We found that TWIST1 is highly enriched at E-box elements of *Dbp* in the presence of TNF (120- fold). In the absence of TNF, CLOCK is 243-fold enriched at the *Dbp* E-box. The knock-down of *Twist1* by siRNA treatment abrogates the enrichment of TWIST1 to the *Dbp* E-box (22-fold) and despite of the presence of TNF enhanced the binding of CLOCK (221-fold) to the E-box (Fig 6A). The overall enrichment at the *Per1* E-box is less pronounced. However, as observed with the *Dbp* E-box treatment with TNF led to an enrichment of TWIST1 compared to CLOCK (63-fold vs. 8-fold) (Fig 6B). In the absence of TNF the ratio is inverted to TWIST1 4-fold, CLOCK 113-fold. Silencing of *Twist1* expression by siRNA increased CLOCK binding at the *Per1* E-box despite of the presence of TNF.

Taken together these results support the hypothesis that TWIST1 induction by TNF competes with CLOCK binding to the E-box.

## Discussion

Dysregulation of clock gene expression by proinflammatory cytokines has been described in fibroblasts, human pancreatic cancer cells, and in leukocytes *in vitro*, and in the liver and SCN of mice with CD40 mediated inflammatory syndrome or upon treatment with lipopolysaccharide (LPS) from gram-negative bacteria, interferon alpha (IFN) or TNF [29–34]. The intracellular pathway responsible for the inhibition of clock genes by cytokines has not yet been identified. Our data identify Twist1 as a TNF induced repressor of the expression of *Per1*, *Per2*, *Per3* and *Dbp*. Upon treatment with TNF and IL-1β NIH-3T3 fibroblasts and HT22 hippocampal neuronal cells show a striking upregulation of *Twist1* and *Twist2*. These data are in agreement with recent reports showing TNF and IL-1β to induce both DNA binding proteins in different types of cells including fibroblasts, macrophages, breast epithelial cells, and various tumor cells [21, 26]. Besides the aforementioned cytokines, also IFNα stimulates *Twist1* expression [26]. However, its induction in macrophages requires treatment of the cells for two days. The delayed response was found to be due to the dependence of *Twist1* induction on IFN induced production of *Axl*. This member of the Tyro 3 kinase family is preferentially expressed in myeloid, neuronal and reproductive system cells. Both the delayed kinetics of *Twist1* induction by IFNα and the cell specificity of *Axl* expression may explain absence of inhibition of clock gene expression when treating NIH-3T3 cells for 4 hr with IFNα.

*Twist1* is involved in development and osteoblast differentiation and has been characterized as an essential player in cancer stem cell generation, tumor invasion and metastasis. The data provided here show *Twist1* to act as a repressor of the transcriptional activity of CLOCK on E-box mediated induction of *Per1* and *Dbp* expression. siRNA against *Twist1* leads to an almost complete prevention from TNF and IL-1β induced inhibition of clock genes. This effect is not shared with effects of siRNA against *Twist2*. Likewise the overexpression of *Twist1*, but not of *Twist2* inhibits clock gene expression in NIH-3T3 cells. Thus *Twist1* is characterized here as the main TNF-induced repressor of clock genes in NIH-3T3 cells. Recently the effect of TNF was shown to be dependent on down-regulation of *Cirbp* [13], which is required for high amplitude expression of clock genes [11, 13]. However, experiments using siRNA against *Cirbp* show that downregulation of *Cirbp* plays only a role in inhibition of clock gene expression at low doses (1 ng/ml) of TNF. At high doses (10 ng/ml) TNF operates in a *Cirbp* independent way [13], the effect of TNF being dependent on induction of *Twist1*.

Many of the target genes of TWIST1 and TWIST2 have multiple E-boxes in their promoters. Induction of transcriptional inhibition by TWIST1 includes blocking of DNA binding and/or the action of other transcriptional activators, binding E-boxes as heterodimers with E-proteins, and recruitment of histone deacetylases [14]. In TNF treated NIH-3T3 cells we find TWIST1 binding to E-box elements in the *Per1* and *Dbp* promoter to be associated with absence of CLOCK protein at the respective sites. These data suggest that by occupying the E-box elements in the *Per1* and *Dbp* promoters TWIST1 inhibits binding of CLOCK. It remains to be seen whether *Twist1* functions as a homodimer or, as reported previously, the effects of *Twist1* may depend on formation of heterodimers with e.g. other class B bHLH proteins [14]. Moreover it can not be excluded that TWIST1 interacts with the master clock genes CLOCK, BMAL1 and/or NPAS2 and thereby hinders their access to the E-box or alters the intrinsic histone acetyltransferase activity of CLOCK, which has been well described to contribute to chromatin-remodeling events implicated in circadian control of gene expression [35]. But generally TWIST binds direct to the E-box to induce or repress transcription [36, 37].

While TGFβ2 shares with TNF and IL-1β its effect to inhibit the expression of *Period* and of the PAR bZip genes *Dbp*, *Tef*, and *Hlf*, but not of *Bmal1* and *Clock* [9], the interference of TGFβ2 with the clock system is found here to be independent of *Twist* (see above). In contrast to TNF and IL-1β, TGFβ2 does not activate *Twist1* expression. In line with absence of *Twist1* activation we find siRNA against *Twist1* not to be protective for the effect of TGFβ2 to inhibit clock gene expression. Previous work showed TGFβ to act on the circadian system by induction of *Dec1* [8]. *Dec1*, another bHLH factor, is induced by CLOCK:BMAL1 heterodimer via the CACGTG E-box in the promoter and, thereafter, suppresses transcription by competing with CLOCK-BMAL1 for the DNA binding. DEC1 upregulation by TGFβ resets the circadian phase of peripheral clocks [38]. The different pathways involved in the negative control of clock gene expression by TNF/IL-1β and TGFβ is interesting from an immunological point of view. While TNF/IL-1β are mainly increased in the acute phase of the immune response, TGFβ is a cytokine, which dampens immune effector mechanisms and mediates repair processes at later stages of inflammatory diseases.

Being induced by proinflammatory cytokines, *Twist1* may be increased in the periphery and / or in the brain in states of immune activation in infectious–and autoimmune diseases. Upregulated *Twist1* may lead to low levels of E-box dependent transcription induced by CLOCK-BMAL1. Since E-boxes are very frequent in the mammalian genome, CLOCK-BMAL1 heterodimers regulate the transcription of many genes besides the clock genes *Per* and *Cry*. Thus the TNF mediated increase of *Twist1* may modulate a multitude of physiological processes. Experimental *in-vivo* evidence in support of the hypothesis would require mice with a deletion of the *Twist1* gene. However, *Twist1* ^-/-^ mice die at E10.5 with increased apoptosis in multiple tissues [15]. Mutations in human *Twist1* lead to the Saethre-Chotzen syndrome, which is associated with severe abnormalities in fusion of the cranial bones and digit defects [39]. Thus cell-type specific inducible deletions of the *Twist1* gene are required to substantiate the hypothesis that *Twist1* transmits signals of the immune system to the clock gene network at the transcriptional level.

The ability of TWIST1 to bind to E-box elements and to compete with CLOCK-BMAL1 mediated activation of *Period* genes and *Dbp* points to a previously unrecognized function of Twist1 and suggests that Twist1 plays a critical role linking the immune and clock systems.

## Supporting Information

S1 Dataset Data of Fig 1. (XLSX)

S2 Dataset Data of Twist1 siRNA and overexpression. (XLSX)

S3 Dataset qPCR Data of time kinetic experiments. (XLSX)

S4 Dataset Twist2 data. (XLSX)

S5 Dataset Reporter gene expression data. (XLSX)

S6 Dataset Data of qChIP experiments in Fig 4. (XLSX)
